# Impacts of natural and human drivers on the multi-decadal morphological evolution of tidally-influenced deltas

**DOI:** 10.1098/rspa.2018.0396

**Published:** 2018-11-07

**Authors:** B. Angamuthu, S. E. Darby, R. J. Nicholls

**Affiliations:** 1 Geography and Environment, University of Southampton, Southampton SO17 1BJ, UK; 2 Engineering and the Environment, University of Southampton, Southampton SO17 1BJ, UK

**Keywords:** tidal delta, morphology, numerical modelling, sea-level rise, polder, cross-dam

## Abstract

The world's deltas are at risk of being drowned due to rising relative sea levels as a result of climate change, decreasing supplies of fluvial sediment, and human responses to these changes. This paper analyses how delta morphology evolves over multi-decadal timescales under environmental change using a process-based model. Model simulations over 10^2^ years are used to explore the influence of three key classes of environmental change, both individually and in combination: (i) varying combinations of fluvial water and sediment discharges; (ii) varying rates of relative sea-level rise; and (iii) selected human interventions within the delta, comprising polder-dykes and cross-dams. The results indicate that tidal asymmetry and rate of sediment supply together affect residual flows and delta morphodynamics (indicated by sub-aerial delta area, rates of progradation and aggradation). When individual drivers of change act in combination, delta building processes such as the distribution of sediment flux, aggradation, and progradation are disrupted by the presence of isolated polder-dykes or cross-dams. This suggests that such interventions, unless undertaken at a very large scale, can lead to unsustainable delta building processes. Our findings can inform management choices in real-world tidally-influenced deltas, while the methodology can provide insights into other dynamic morphological systems.

## 1. Background

River deltas are iconic geomorphological features that provide ecosystem goods and services that support the lives and livelihoods of hundreds of millions of people worldwide [1,2]. However, as key climate change hot spots the world's deltas are currently facing a major sustainability crisis. Specifically, many of the world's deltas are at risk of ‘drowning’ due to rising relative sea levels, declining sediment supplies and the adverse impacts of human agency. On this latter point Syvitski *et al*. [3] reported that the recent intensity of human interventions across the world's deltas is now so high that the morphodynamic evolution of many deltas can no longer be considered natural, placing a number of deltas in greater danger of inundation due to reductions in floodplain sedimentation, accelerated subsidence and sea-level rise. Particular challenges are associated with the mega-deltas that are the specific focus of this study. The world's largest deltas are massive geomorphological features which merit attention through their sheer scale—the Ganges-Brahmaputra-Meghna (GBM) delta alone covers more than 100 × 10^3^ km^2^ and is home to more than 100 million people [4–6]. Some of the largest cities in the world, such as Shanghai, Kolkata, Dhaka and Cairo, are located on mega deltas. With the environmental stresses on deltas expected to increase, both as a result of ongoing climate change as well as increasing population, urbanization and socio-economic change [5,7,8] the human impacts on the world's deltas are likely to increase through the twenty-first century. This raises the question of how the morphology of deltas might evolve in the future and how humans may better manage deltaic ecosystems.

For all these reasons, a detailed understanding of the ways in which delta morphology adjusts to environmental changes and anthropogenic intervention is an essential pre-requisite for the sustainable management of deltas. Recently, progress has been made in understanding the formation of deltas under the influence of waves, tides, and rivers and for a variety of different sediment characteristics [9–15] and processes of deltaic land building and restoration [16–18]. However, our ability to simulate delta response to natural and human-induced environmental change and management interventions remains limited [19,20] by two key challenges.

The first of these challenges concerns the problem of scale. As noted above, many of the most significant issues facing deltas and their inhabitants are in the vulnerable mega-deltas where the high population densities place large numbers of people at risk. However, the kinds of process-based simulation models that are necessary to deliver reliable predictions of morphodynamic response to environmental change are often computationally burdensome. Consequently, until recently computational expense has meant that it has been prohibitive to implement process-based models across the large spatial and temporal domains that are necessary to characterize response dynamics on the world's major deltas.

The second challenge relates to the point that much of our existing understanding of delta morphodynamics comes from wave-dominated systems, which globally are the most common type. However, there appears to be a systematic bias in the geographical distribution of the large deltas that are most vulnerable to environmental change, with more than half of the 23 large deltas reported by Ericson *et al*. [1] being strongly tidally influenced. Indeed, some 27 of 51 deltas studied to understand the influence of humans on the morphodynamics of deltas [6] have a tidal range greater than 2 m. It is therefore the case that the prior focus on wave-dominated deltas has led to a relative paucity of our understanding of tidally-influenced deltas [9,20–24].

To address the issues outlined above, this paper aims to *determine how tidal delta morphology evolves over multi-decadal timescales and under multiple drivers of environmental change.* Specifically, we categorize individual drivers of environmental change into three broad classes: catchment-scale changes (water and sediment fluxes supplied from the feeder catchment upstream), global-scale changes (sea-level rise), and local human interventions (with a specific focus here on common engineering interventions such as polder-dykes and cross-dams) and then we evaluate their impact on deltaic morphodynamic response. To address this aim we explore a number of specific research questions as follows:

(1) What is the role of catchment and global-scale drivers of change in forcing the morphodynamic response of tidally-influenced deltas, and especially (i) how do variations in fluvial water and sediment discharges supplied from the feeder catchment upstream affect tidal delta morphology, and (ii) what is the role of relative sea-level rise (RSLR) in delta building?
(2) What is the role of typical human interventions in affecting delta morphodynamic response? What is the impact of common interventions such as building (i) polder-dykes and (ii) cross-dams, on tidal delta morphology?
(3) What is the morphodynamic response of deltas under multiple interacting drivers of change and how does the response of deltas differ when these drivers act in combination or isolation?

These research questions have been set to address the need to derive a greater understanding of the factors influencing the morphological evolution of an idealized tidal delta (albeit the idealized delta investigated herein is similar to the Ganges-Brahmaputra-Meghna (GBM) mega-delta). As the first attempt to address these issues for a large-scale tidally influenced delta, the insight gained from this work may be useful in aiding the development of delta management strategies for tidally influenced deltas globally.

## 2. Methodology

### (a) Approach

To address the research questions defined above, we employ a process-based, two dimensional numerical model built using the Delft3D modelling software package [25] to undertake a range of simulations designed to elucidate our understanding of the impact of environmental changes on delta morphology over multi-decadal timescales. It is important to note that our focus on large deltas presents specific challenges in terms not only of the significant computational burden required to run multiple model simulations over many years and across large spatial domains, but also in the need to parametrize the model appropriately. Unfortunately, data availability in large deltas, particularly those located in the Global South, is frequently highly constrained and this precludes building a model that seeks to replicate precisely a prototype mega-delta. For these reasons we instead develop a set of model simulations that seeks to replicate the essential attributes of an idealized tidally-dominated delta (in this case, based loosely on the GBM delta), if not its actual topography and biophysical characteristics, so as to enable qualitative assessments of generic system response to various forcings [26]. Thus, the insights garnered from this approach are likely to be transferable in broad terms to the GBM delta, as well as other tidally dominated deltas. This approach follows a few prior studies that have investigated the long-term morphodynamics of, for example North Sea tidal basins [15,27–30] and the Yangtze River estuary [14]. Nevertheless, as far as we are aware, our focus on tidally-influenced deltas means that this study represents the first attempt to model an idealized geomorphological system over such a large spatial scale.

### (b) Model initialization and boundary conditions for the baseline run

**Figure 1.**
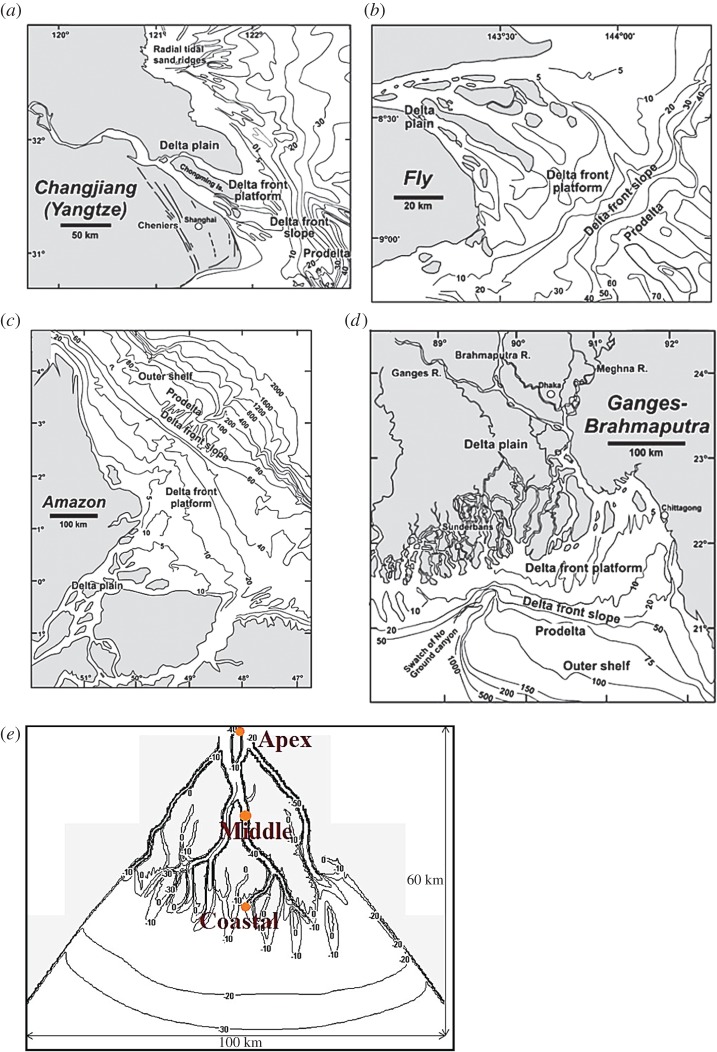
Four major tide-dominated delta systems: (*a*) Changjiang/Yangtze, (*b*) Fly, (*c*) Amazon, (*d*) Ganges-Brahmaputra, and (*e*) Overview of the idealized tidal delta illustrating the initial conditions for the experimental simulations undertaken herein ((*a–d*) taken from Goodbred & Saito [31]). Note: Orange circles in subplot (*e*) show the locations of model results presented in figure 3. (Online version in colour.)

This section describes the modelling approach applied to produce a delta analogous to the eastern part of the real GBM delta in the 1940s (figure 1). The 1940s was selected as a reference state because at this time the GBM delta was in a relatively pristine condition, at least compared to subsequent changes [32]. An essential characteristic of tide-dominated deltas is that they all tend to exhibit funnel-shaped river mouths, well-developed channel bars and islands [31], as modelled in this study (figure 1). These initial morphological conditions are similar in planform to those within the GBM delta, but the actual initial morphological conditions used in the numerical experiments were established by allowing the simulated delta to morphologically evolve during a model ‘spin-up’ period designed to generate the experimental sub-aerial delta (figure 1*e*). Details of the model set-up and parameters employed are in the electronic supplementary material.

The initial condition for each numerical experiment (see §2c for details of the numerical experiments undertaken) was similar to meaningful physical properties of the actual GBM delta wherever possible. Thus, the hydrodynamic boundary of the model at the downstream limit of the domain is forced by a semi-diurnal tide with a range of 0.75 m. The tidal range is uniform across the boundary and constant over time, which is a simplified representation of the water level in the Bay of Bengal, but ignoring the tidal constituents and the effect of spring-neap tidal cycles. Sediment transport at the downstream boundary was set using an equilibrium sediment concentration. This means that sediment concentration at this boundary adapts to the local flow conditions near instantaneously.

The river discharge imposed at the upstream boundary was set based on the actual fluvial discharge of the Ganges and Brahmaputra Rivers entering the delta at the top of the lower Meghna River. Specifically, following the methodology of Thorne *et al*. [33] and Biedenharn *et al*. [34] we estimated the river's dominant discharge, giving a value of 65 000 m^3^ s^−1^, which is approximately equivalent to the flow with a return period of 1 year. The percentage of time the dominant discharge flow is equalled or exceeded is 7%, or 26 days. Sediment transport rates at the upstream boundary (for the dominant discharge) were set to 130 mg l^−1^ for the sand fraction and 120 mg l^−1^ for the silt fraction as estimated from empirical relationships developed by the Bangladesh Water Development Board (BWDB) that link sediment concentration for each size fraction to water discharge and using data for the period 1992 to 2012.

Hydrodynamic and transport processes vary over hours to days, whereas morphological changes occur over much longer periods [35]. This means that bed levels in the model are updated at time intervals equal to the product of the morphological acceleration factor (MORFAC) and the hydrodynamic time step, thereby enabling quicker computation while maintaining mass conservation. Generally, a MORFAC value of up to 500 is recommended for tidal conditions [35]: here a value of 30 was used.

After a simulation period of 60 years, the rate of bed level change in the modelled delta area reduced significantly. This indicates that the morphological evolution of channels and the rate of aggradation and progradation of the delta slowed to reach a condition approaching morphodynamic equilibrium. It also implies that hydrodynamic equilibrium is reached in the delta area. The terminal distributaries and the mouth bars evolved in a manner typical of tide-dominated deltas [21]. For example, each mouth bar formation resulted in trifurcation (figure 1*e*). The extent of the model also permits proper exchange of sediment between the sea and delta, which is consistent with the study of van der Wegen *et al*. [29]. The tidal range within the modelled delta ranges from 2.5 to 3 m and channel depths range from 30 to 50 m. The maximum water flow velocity in the channels is 1.65 m s^−1^ for the outflow and 0.65 m s^−1^ for incoming flows. The modelled bathymetry and the tidal flow velocities are, therefore, broadly representative of the actual values observed within the Meghna estuary [36,37].

**Figure 2.**
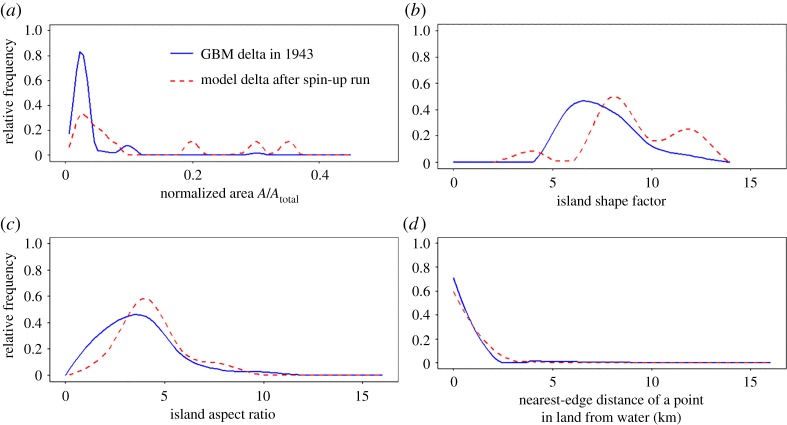
Probability density functions of selected delta morphology metrics comparing the morphological status of the GBM delta as observed in 1943 (Source: CEGIS) with the idealized tidal delta modelled at the end of the ‘spin up’ simulation undertaken herein. (*a*) Island size normalized to total island area, (*b*) island shape factor, (*c*) island aspect ratio, and (*d*) nearest edge distance of a point in land from water. (Online version in colour.)

The extent to which the analogue delta is an appropriate representation of a tidally-dominated delta, using the GBM delta as a reference, was assessed by comparing selected simulated and observed delta metrics. First, the ratio of cumulative distributary channel width to river width reflects the power of fluvial and tidal energy [6]. Here, the cumulative distributary channel width is a function of bank-full discharge and is positively influenced by tides. This ratio for the modelled delta (figure 1*e*) was found to be (56 km/3 km) 18.7, compared to the observed (1943) value of 28.5 [6]. The following delta metrics: island area, island shape factor (indicates the degree of drainage within islands), island aspect ratio (indicates the degree of elongation of the islands) and the nearest-edge distance of a point in land to the water all showed a good match between the simulated idealized delta and the real world GBM delta (figure 2). This indicates that the delta produced after the ‘spin-up’ period (figure 1*e*) is a representative initial condition.

### (c) Numerical experiments: delta response to environmental change

Through a set of carefully designed numerical experiments, we explored the response of deltaic morphodynamics to a range of external drivers over multi-decadal timescales. A base case scenario was defined by employing the same forcing used during the spin-up period with no environmental change (i.e. with zero rate of relative sea-level rise and with no change in the imposed water and sediment discharges) or anthropogenic disturbances. In the sub-sections below details of the specific scenarios employed are provided. Note that all the simulations were performed on the University of Southampton supercomputer, Iridis4, with typical run times of around 2.5 weeks per simulation. This emphasizes that the computational burden required in such studies is large enough to have been prohibitive until recently, and these results represent a significant innovation.

#### (i) Environmental change scenarios: variations of fluvial discharge, sediment load and sea-level rise

Many of the large rivers that feed the world's mega-deltas have in recent decades experienced, or are likely to experience in the coming years, environmental change that can induce significant changes in the water and sediment discharges reaching delta apices. Common causes of such changes include upstream damming [3,38–40], soil conservation and sediment control programmes [39], land clearance and subsequent land use activities such as agricultural practices [41], inter basin water transfers [42], and changes in climate [43,44]. The potential impacts of changes in these drivers on delta morphology are explored here using a matrix of scenarios in which different combinations of water (*Q*_w_) and sediment (*Q*_s_) discharge are applied. Specifically, we vary both *Q*_w_ and *Q*_s_ from relatively low to relatively high values, producing a combination of nine scenarios (see table 1 for details). In the case of the water discharge, the range is defined by varying *Q*_w_ in the range −53% to +31% of the dominant discharge of *Q*_w_ = 65 000 m^3^ s^−1^, which is consistent with the range of peak floods observed on the real GBM delta [45]. The sediment discharge, *Q*_s,_ was varied in the range −50% and +45% of the dominant discharge of *Q*_s_ = 1.48 m^3^ s^−1^. This range of values is based on the sediment flux reduction over the course of the last century as estimated by Syvitski *et al*. [3] and an upper limit based on future increases (at the end of the current century) in sediment flux for the Ganges and Brahmaputra under the influence of anthropogenic climate change as projected recently by Darby *et al*. [43].

**Table 1.** Overview of numerical experiments in which imposed water and sediment discharges are varied.

		water discharge (*Q*_w_)		
		low (*Q*_w_ = 32 500 m^3 ^s^−1^)	medium (*Q*_w_ = 65 000 m^3 ^s^−1^)	high (*Q*_w_ = 97 500 m^3 ^s^−1^)
sediment discharge (*Q*_s_)	starved	*low flow, sediment starved*	*medium flow, sediment starved*	*high flow, sediment starved*
	sand: *Q*_s_ = 0.80 m^3 ^s^−1^ or 65 mg l^−1^			
	silt: *Q*_s_ = 0.74 m^3 ^s^−1^ or 60 mg l^−1^			
	medium	*low flow, sediment medium*	*base case (medium flow, sediment medium)*	*high flow, sediment medium*
	sand: *Q*_s_ = 3.19 m^3 ^s^−1^ or 130 mg l^−1^			
	silt: *Q*_s_ = 2.94 m^3 ^s^−1^ or 120 mg l^−1^			
	rich	*low flow, sediment rich*	*medium flow, sediment rich*	*high flow, sediment rich*
	sand: *Q*_s_ = 7.17 m^3 ^s^−1^ or 195 mg l^−1^			
	silt: *Q*_s_ = 6.62 m^3 ^s^−1^ or 180 mg l^−1^			

Studies by Ericson *et al*. [1], Syvitski *et al*. [3], Nicholls & Cazenave [46], Saito *et al*. [47], Islam *et al*. [48], Szabo *et al*. [4], and Tessler *et al*. [2] have all also highlighted the increased vulnerability of deltaic ecosystems and human populations due to ongoing and future sea-level rise. However, the processes by which large tidally-influenced deltas respond to sea-level rise remains unknown [49]. We therefore designed a simple set of simulations in which the delta response to varying rates of RSLR is considered. Specifically, based on the sea-level rise estimates of Church [50], Nicholls *et al*. [51] and Pethick & Orford [52] the rate of relative sea-level rise was varied in the range 0–20 mm yr^−1^ by increasing the height of the tidal water level boundary conditions.

#### (ii) Anthropogenic disturbance scenarios: impacts of polder-dykes and cross-dams

The populous nature of many of the world's large deltas, combined with their rich resources, means that investments in a variety of infrastructure projects, frequently designed to afford flood protection and land reclamation for agriculture and other uses, are common. Typical measures include polderization (flood embankment enclosure of land or islands) and the building of cross-dams (fixed structures that permanently close off a river mouth or estuary). However, the influence of these structures on delta morphology, in particular in relation to the propagation of non-local disturbance effects across large deltas, remains unclear. This represents a significant limitation when the use of such structures is likely to increase in the future as developing nations seek ways to encourage economic growth in delta regions [5,53–55]. Flood embankments within the coastal area of deltas do not allow fluvial and tidal water discharges to flood the delta plains, but this use of embankments may also induce sediment starvation inside polder-dykes [56,57]. The effect of the consequent *excess* of sediment on delta morphodynamics *outside* the polders also remains unknown [58]. To investigate these potential effects, we designed two additional model scenarios investigating the effects of: (i) polder-dykes; and (ii) cross-dams. To represent poldered areas in the model, flood embankments were simulated as thin dams, that is as impervious structures of infinite height, meaning that the enclosed areas were not allowed to flood or erode. Cross-dams were represented in the model simulations by completely blocking (sudden closure of the channel) the flow in the relevant distributary, which has the effect of increasing water and sediment flow through the other distributaries.

#### (iii) Integrated response scenarios: the combined effects of environmental change and anthropogenic disturbance

In reality, variations in the individual driving factors outlined in the experiments above are unlikely to occur in isolation [58–62]. Therefore, in this third and final set of numerical experiments we covaried the various controlling factors to represent more likely morphodynamic response pathways, as set out in table 2. However, other experimental scenarios could be investigated in future research.

**Table 2.** Numerical experiments in which scenarios combining environmental change (varying fluvial and sediment discharges and relative sea-level rise) with anthropogenic disturbances (polders and cross-dams) are explored. Highlighted texts show the change in driver and parameter values.

s. no.	combined scenarios	driver and parameter values
1	**P+CD**	combination of **polder-dykes and cross-dams**
2	P+CD+**2mSLR**	combination of polder-dykes, cross-dams, and **2 m sea-level rise after 100 years at the rate of 20 mm yr^−1^**, sediment medium (sand: *Q*_s_ = 3.19 m^3 ^s^−1^ or 130 mg l^−1^; silt: *Q*_s_ = 2.94 m^3 ^s^−1^ or 120 mg l^−1^)
3	P+CD+1mSLR+medium flow	combination of polder-dykes, cross-dams, 1 m sea-level rise after 100 years at the rate of 10 mm yr^−1^, and medium flow (*Q*_w_ = 65 000 m^3 ^s^−1^), **sediment rich** (sand: *Q*_s_ = 7.17 m^3 ^s^−1^ or 195 mg l^−1^; silt: *Q_s_* = 6.62 m^3 ^s^−1^ or 180 mg l^−1^)
4	P+CD+1mSLR+medium flow, **sediment starved**	combination of polder-dykes, cross-dams, 1 m sea-level rise after 100 years at the rate of 10 mm yr^−1^, and medium flow (*Q*_w_ = 65 000 m^3 ^s^−1^), **sediment starved** (sand: *Q*_s_ = 0.80 m^3 ^s^−1^ or 65 mg l^−1^; silt: *Q*_s_ = 0.74 m^3 ^s^−1^ or 60 mg l^−1^)
5	P+CD+1mSLR+**high flow**, sediment medium	combination of polder-dykes, cross-dams, 1 m sea-level rise after 100 years at the rate of 10 mm yr^−1^, and **high flow (*Q*_w_ = 97 500 m^3 ^s^−1^)**, sediment medium (sand: *Q*_s_ = 3.19 m^3 ^s^−1^ or 130 mg l^−1^; silt: *Q*_s_ = 2.94 m^3 ^s^−1^ or 120 mg l^−1^)

## 3. Results

**Figure 3.**
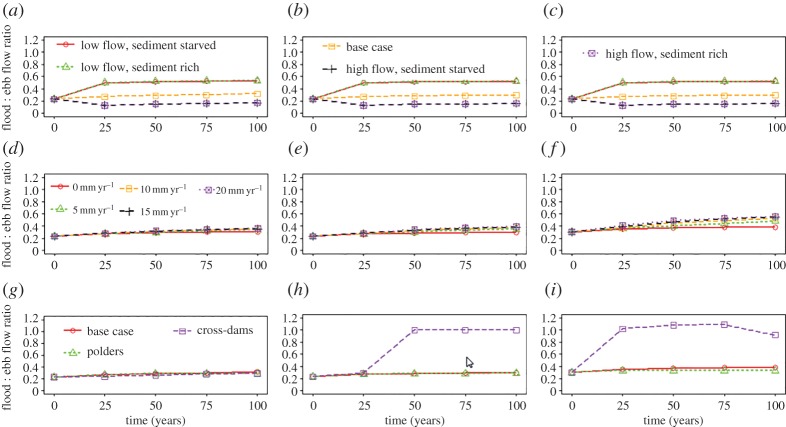
Spatial and temporal variations of the flood : ebb flow ratio for the numerical experimental scenarios investigated herein. The delta apex (left hand column, subplots *a*, *d* and *g*), mid-delta (middle column, subplots *b*, *e* and *h*) and coastal locations (right hand column, subplots *c*, *f* and *i*) investigated in these simulations are illustrated by the orange circles in figure 1*e*. Subplots show the simulated flood : ebb flow ratio where (*a–c*) are for varying water and sediment discharges as defined in table 1; (*d–f*) are for varying rates of sea-level rise (in the range 0–20 mm yr^−1^); and (*g–i*) are for scenarios with human interventions: polders and cross-dams. (Online version in colour.)

In this section results from the model simulations outlined above are presented and discussed. The simulated delta responses are analysed firstly in terms of the delta hydrodynamics, as represented here by the flood : ebb flow ratio. The flood : ebb flow ratio (as measured using the tidal discharge as the index of flow) is widely recognized as a key metric because it affords a good representation of the tidal asymmetry (note that tidal asymmetry is defined by the difference in intensity and duration of the ebb and flood tidal flows. For ebb-dominated residual flows, the flood : ebb ratio is less than 1 and for flood-dominated flows it is greater than 1.). For all scenarios shown in figure 3, time zero represents the initial condition of the numerical experiment, so that any change in upstream river discharge has not yet occurred. Second, we focus on morphodynamic changes as represented by various morphological metrics [63] including island area (indicates sub-aerial delta area), island aspect ratio (indicates the degree of elongation of the islands in the delta), island shape factor (indicates the degree of drainage within the islands of the delta as estimated by the ratio of wetted perimeter to the square root of island area) and nearest-edge distance (defined as the shortest, straight line distance of a point in land to the nearest water), simulated patterns of erosion and accretion over the sub-aerial and sub-aqueous delta with respect to a mean sea level (MSL) of 0 m, and rates of net aggradation and progradation.

### (a) Base case scenario

**Figure 4.**
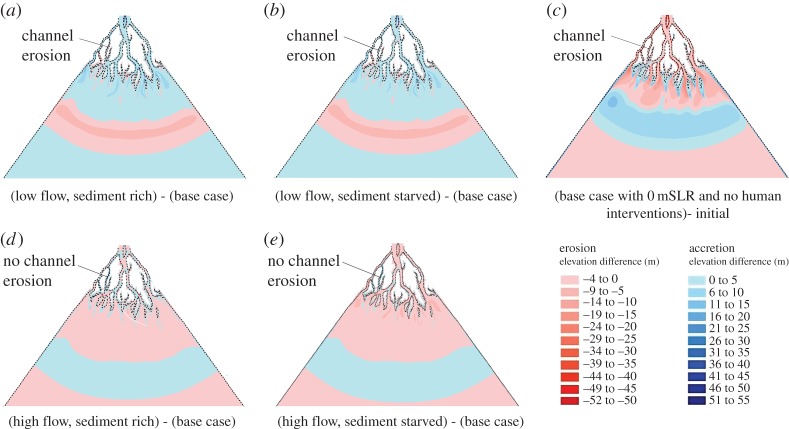
Spatial patterns of accretion and erosion over the modelled delta after 100 years of simulation. Subplots (*a*,*b*,*d*,*e*) illustrate scenarios in which the ratio *Q*_w_/*Q*_s_ is varied from relatively low (sediment rich scenarios) to relatively high (sediment starved scenarios) with respect to the base case scenario of *Q*_w_/*Q*_s_ = 1 shown in subplot (*c*). Dashed line indicate the Sub-aerial delta at the initial condition. Mean sea level (MSL) is assumed to be 0 m. *Q*_w_, water discharge; *Q*_s_, sediment discharge.

**Figure 5.**
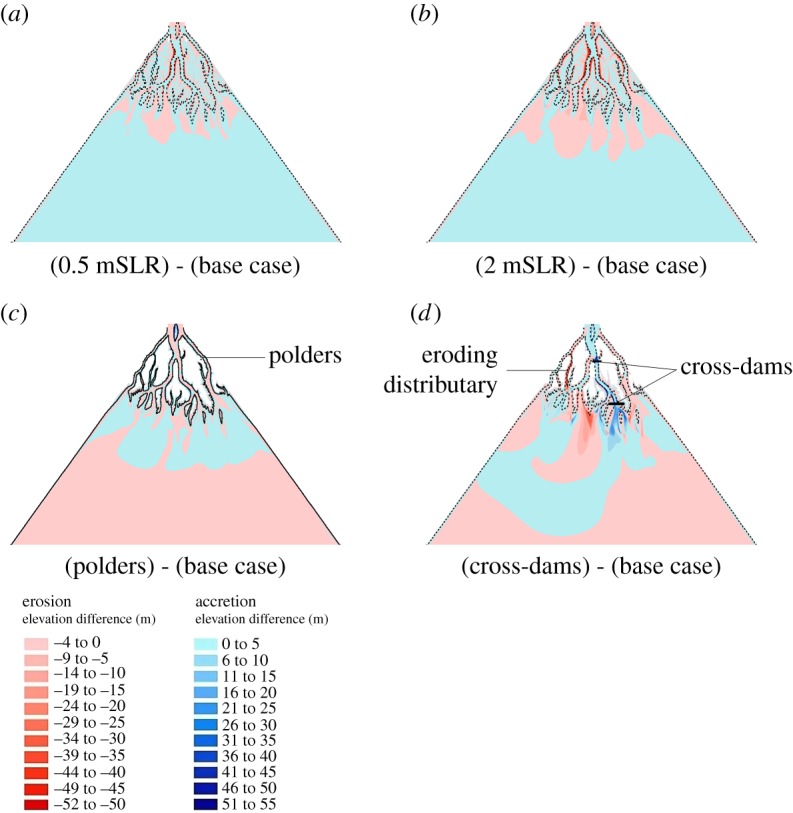
Spatial patterns of accretion and erosion over the modelled delta after 100 years of simulation. Subplots (*a*,*b*) represent scenarios with 5 and 20 mm yr^−1^ rates of sea-level rise, respectively; while subplots (*c*,*d*) represent scenarios with polder-dykes and cross-dams, respectively. Dashed line indicate the sub-aerial delta at the initial condition. Mean sea level (MSL) is assumed to be 0 m. SLR, sea-level rise.

**Figure 6.**
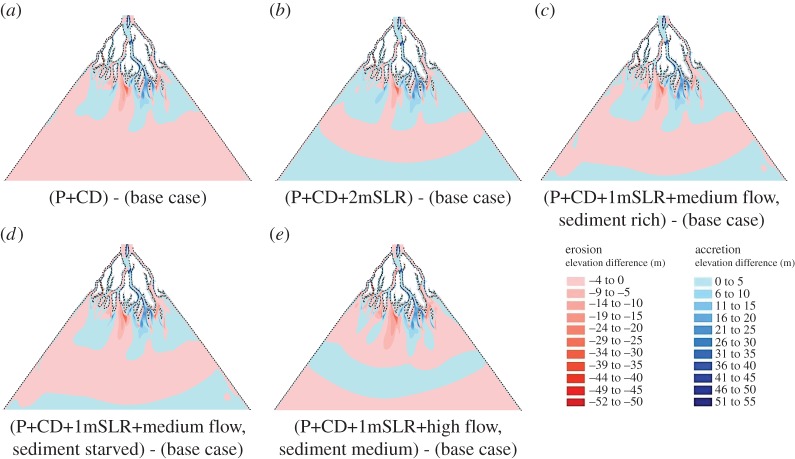
Spatial patterns of accretion and erosion over the modelled delta after 100 years of simulation. Subplots (*a*–*e*) represent the integrated response scenarios, with parameter values as listed for scenarios 1, 2, 3, 4 and 5 as detailed in table 2, respectively. Dashed line indicate the Sub-aerial delta at the initial condition. Mean sea level (MSL) is assumed to be 0 m. For the purpose of clarity, the locations of the polders and cross-dams locations are not indicated on the subplots. CD, cross-dams; P, polders; SLR, sea-level rise.

**Figure 7.**
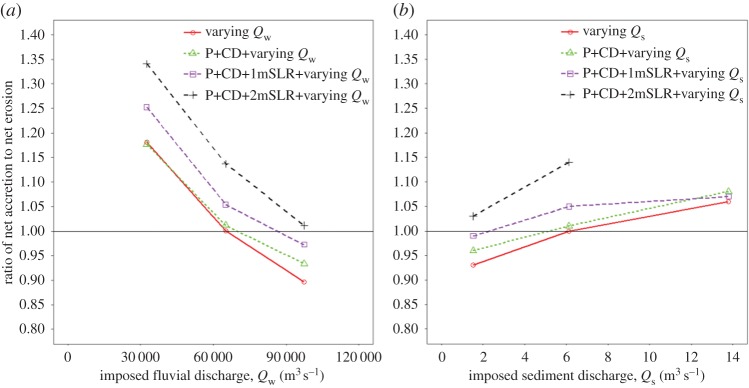
Ratio of net accretion to erosion in the ideal tidal delta after 100 years of model simulation and for a range of intervention scenarios as a function of: (*a*) variations in the imposed fluvial water discharge; (*b*) variations in the imposed fluvial sediment discharge. CD, cross-dams; P, polders; SLR, sea-level rise. *Q*_w_, water discharge; *Q*_s_, sediment discharge. (Online version in colour.)

The results of the base case scenario, which is the scenario with no environmental changes (i.e. the rate of relative sea-level rise is zero and there are no changes over time of water or sediment discharge) or anthropogenic disturbances (no engineering interventions), are now outlined to provide a basis for comparison with the other numerical experiments discussed below. In this scenario the flood : ebb flow ratio ranged between 0.2 and 0.4, showing that the residual flow is ebb dominated. However, over the course of the 100-year simulation period the initial flood : ebb flow ratio gradually increased (by 30%) at many locations throughout the delta (including sites near the apex, middle, and close to the mouths of the distributary channels, as illustrated in figure 3) as a hydraulic response to the ongoing morphological adjustments. These morphodynamic adjustments are shown in figures 4–6 (figure 4*c* for the baseline scenario), which shows the plan view pattern of net erosion (indicated by red shades) and accretion (indicated by blue shades) at the end of the 100-year simulation period. Zones of erosion and accretion (calculated with respect to MSL of 0 m) were located throughout the delta domain, but most of the accretion, both on the sub-aerial delta and within the distributary channels, was concentrated closer to the seaward of the delta, with some accretion also located at the heads of the intra-island channels. The dark red patches, indicating erosion of channel banks along the sub-aerial delta, were more extensive under conditions of relative local sediment starvation, with the main locus of the erosion in the baseline scenario located in the landward part of the delta, and on the channel and sea floor bed seawards. However, the ratio of net accretion to erosion (i.e. the ratio of the total volume of sediment accretion to total volume of erosion over the sub-aerial and sub-aqueous delta) at the end of the 100-year simulation period is equal to 1 (figure 7*a*), which shows that the base case scenario generates a delta in overall dynamic morphologic equilibrium.

### (b) Environmental change scenarios

#### (i) How do fluvial water and sediment discharges affect tidal delta morphology?

Here we investigate our first research question about *how variations in the supply of freshwater discharge (Q_w_) and fluvial sediment (Q_s_) from the upstream catchment affect delta morphodynamics*. This question is explored by varying the relative proportions of *Q*_w_ and *Q*_s_ imposed at the upstream boundary of the model (table 1), while holding all other parameters and boundary conditions at their baseline values. The relative strength between the flood and ebb flow of the tidal cycle is strongly influenced by the fluvial water discharge, as shown in figure 3. In the simulations conducted herein, the ratio of the flood to ebb flow is 0.5 for the low river discharge and 0.1 for high river discharge respectively, meaning that a factor of 3 variation in fluvial water discharge induces a factor of 5 variation in the flood to ebb flow ratio. Also noteworthy is that variations in the imposed supply of fluvial water discharge affect the phase lag between the water level, velocity, residual flow, and sediment transport. However, tidal asymmetry varied with water discharge, but not with sediment discharge because the morphodynamic response in the delta for scenarios of varying sediment discharge was not significant enough to influence the hydrodynamics.

Variations in the relative proportions of *Q*_w_ and *Q*_s_ also affected the pattern of simulated net erosion and accretion across the delta. As expected, the model simulation for the low flow, sediment rich scenario produces a much greater amount of accretion and much less erosion over the delta, relative to the base case scenario (figure 4*a*). In contrast, for the scenario in which flow is increased relative to sediment supply (figure 4*e*), there is much more accretion located in front of the prograding islands at the delta front and a greater extent of accretion within the tidal floodplain, but with much greater erosion of the island banks, as well as on the beds of the distributary channels and the offshore basin. In effect this is a sediment starved scenario, however, greater accretion is observed because of the rapid expansion in the cross-sectional area when the confined ebb flow enters the sea [10] and greater extent of inundation in the tidal floodplain. The spatial and temporal change of accretion and erosion over the delta can be summarized numerically by the ratio of accretion and erosion as shown in figure 7. Figure 7 shows that the import of sediment into the delta increases with decreasing water discharge (figure 7*a*) [64] and/or increasing sediment discharge (figure 7*b*), with the accretion : erosion ratio decreasing from 1.18 to 0.89 (25% decrease) in response to a threefold increase in water discharge, while increasing from 0.94 to 1.06 (12.7% increase) for a threefold increase in sediment discharge. This indicates that the volume of accretion and erosion over the delta is sensitive to the input rates of (and the balance between) fluvial water and sediment discharge.

Note that channel erosion on the bank of an island in the north-western part of the model domain (location indicated by the label ‘Channel Erosion’ in figure 4*a–c*), causes the island to split into two in the low and medium flow discharge scenarios, due to the changes in residual flow within this distributary (increasing from zero to 3000 m^3^ s^−1^ for the base case scenario). But, no channel erosion was simulated in the high discharge scenario (figure 4*d*,*e*) as the stronger ebb flow in the adjoining distributaries did not alter the residual flow in this location. This result is broadly consistent with the finding of Edmonds *et al*. [65] that the number of distributaries is a function of river discharge. But, in the present study the number of distributaries increases with decreasing river discharge and is different from Edmonds *et al*. [65], where the number of distributaries increases with increasing river discharge. This interesting result arises because of the tidal dominance in the current simulations: In locations where ebb flows are dominant, the number of distributaries decreases, but where the flood flows dominate the number of distributaries increases. As explained by Kleinhans *et al*. [66], within deltas, when flow is dominated by the tides, the bifurcations become confluences.

#### (ii) What is the role of relative sea-level rise in tidal delta morphology?

Our second research question, namely *on the effects of relative sea-level rise on delta morphodynamics*, was explored by imposing a RSLR in the range 0–20 mm yr^−1^ at the seawards boundary of the model. The major impact of increasing RSLR was to induce a modest (albeit increasing seawards) increase in the flood : ebb flow ratios simulated over the delta (figure 3*d–f*). Specifically the simulation results shown in figure 3*d–f* highlight how there is a gradual increase in the dominance of the tidal discharge against the fluvial discharge as RSLR increases, albeit it must still be recognized that in all the simulations the ebb flow still dominates the flood flow as the flood : ebb flow ratio remains less than 1. The effect of the increased backwater due to sea-level rise increases the flood risk on the deltaic islands and reduced flow velocities. With the flow area over the delta, the depth of flow within the distributary channels, and the depth and extent of flooding over the tidal floodplain all increasing with increasing RSLR, there is an overall increase in accommodation space [20] that induces location-dependent sediment accretion or erosion, depending on the local balance between sediment transport capacity and supply. For example, figure 5*a*,*b* clearly shows the difference in the patterns of simulated erosion (red shades) and accretion (blue shades) between the base case scenario and the scenarios of 5 and 20 mm yr^−1^ of sea-level rise, respectively. These data show that as RSLR was increased, the deposition of sediment on the channel bed and land increased. However, at the same time, the submergence or erosion of island banks and the land surface also increased. Overall, land tends to be lost on the landward side, whereas aggradation and progradation are focused at the delta front for all the RSLR scenarios.

An interesting facet of the simulations is that the model results exhibited a tendency for the zone of maximum accretion to be located at the point inland that is farthest from the channel, alongside a tendency to produce feather-shaped patterns of accretion over the sub-aerial delta surface. The width [64] and depth of the channels also increased with increasing RSLR, as shown in figure 5*a*,*b*. The upstream propagation of bed aggradation also increased with increasing RSLR, as shown in figure 5*a*,*b*, because of the increased backwater effect. Even though this process is similar to the one triggered by the mouth bar formation [67], no intra-delta lobe avulsion was apparent in our simulations. For all the (5, 10, 15 and 20 mm yr^−1^) RSLR scenarios explored here, net erosion was marginally greater than net accretion after 100 years of the simulation. Hence, overall net accretion/erosion did not change significantly with increasing RSLR, at least under the imposed water and sediment discharges investigated.

The response of the idealized tidal delta to increasing rates of sea-level rise simulated here is similar to that discussed by van der Wegen [28] for tidal channels, with rapid inundation of lowland areas combined with growth of upland areas over the deltaic islands. Similarly, the planform evolution of the deltaic islands and distributaries illustrated in this work is consistent with the empirical findings of Stefanon *et al*. [68] who demonstrated that the change in the tidal prism induced by sea-level rise strongly influences channel cross-sectional areas, tidal channel network structure and drainage density. This consistency is interesting in that our simulations were undertaken at a much larger spatial scale and included the tidal interaction with fluvial discharge. The shape of the evolved islands and distributaries is more prominent than in the tidal basin evolution modelled by van der Wegen [28] and Stefanon *et al*. [68] as the river flow also contributes to the tidal prism in tide-dominated deltas.

### (c) Anthropogenic disturbance scenarios

Here we explore the impact of engineering structures commonly employed in the world's major deltas, with a specific focus on polder-dykes and cross-dams.

#### (i) What is the impact of building polder-dykes on tidal delta morphology?

Our model simulations show that the simulated polders prevented the inundation of the tidal floodplains, thereby decreased the tidal prism. Note that in the model scenario with polder-dykes, water was not allowed to overtop or breach the polder-dykes, and the poldered banks were assumed non-erodible. The introduction of polder-dykes led to a reduction in the flood : ebb flow ratio (figure 3*g–i*) indicating an increase in tidal asymmetry across the delta. The duration of the flood flow phase was only about 60% of the ebb flow, resulting in ebb-dominated flow. Consistent with the findings of Pethick & Orford [52] and Elias & van der Spek [69] our simulations show that the tidal asymmetry increased as a result of reduced frictional damping and an increased rate of channel convergence in the presence of the polder-dykes. Furthermore, their introduction caused a shift in tidal phase due to all the water remaining within the distributaries (rather than flooding the poldered areas) during the flood phase. The increased volume of water and greater water depth in the distributaries, along with the reduced bed friction, therefore increased the sediment transport capacity of the flow in the poldered scenario.

Figure 5*c* illustrates the difference in simulated morphology between the polder-dykes scenario and the base case (no polder-dykes) scenarios after 100 years of the simulation period. It is evident that polderization induces greater erosion of the channel bed (red shades) within the distributary channels and deposition (blue shades) on the seaward side of the prograding islands. Here the loss of tidal prism due to the embankments is offset by an increase in channel cross-sectional area. Similar to the observations by Falcini *et al*. [70], sediment was also discharged farther into the sea in the model simulation with polder-dykes. The poldered areas of the prograding islands at the delta front were, therefore, relatively deprived of sediment. No bank erosion was predicted in the scenario with polder-dykes but instead accretion was simulated at the head of intra-island channels, as also observed in Wilson *et al*. [71], as the excess water and sediment stays within the channels. This additional water also has the effect of increasing the sediment transport capacity of the flows within the distributary channels. This, in turn, leads to greater bed erosion of the major distributary channels and the increased sediment volumes conveyed in the distributary channels are discharged at the mouths of these distributaries, in turn causing more sediment deposition at the delta front than in the base case scenario. Overall the polder-dykes increased the net accretion as the ratio of accretion to erosion is marginally greater than 1, mainly due to the prevention of island bank erosion in this scenario.

#### (ii) What is the impact of building cross-dams on tidal delta morphology?

Our model simulations (figure 5*d*) show that the introduction of cross-dams had the effect of blocking the flow through the local distributary on which the dam is located, thereby causing a diversion of water and associated backwater effects over the rest of the delta. Importantly this propagates non-local effects through the delta due to the altered flow pattern. Specifically, the reductions of flow velocities in the distributaries where the cross-dams are located induced increased water discharges and flow velocities in distributaries elsewhere. The simulated cross-dams significantly altered the value of the flood : ebb flow ratio, such that the flood and ebb flows were equalized in the distributaries (figure 3*h*,*i*) with cross-dams. Elsewhere, the value of the flood : ebb flow ratio decreased. Water discharges within the eroded distributary (northwest direction, see figure 5*d*) increased by a factor of between six and nine times in the cross-dam scenario, when compared to the base case scenario.

**Figure 8.**
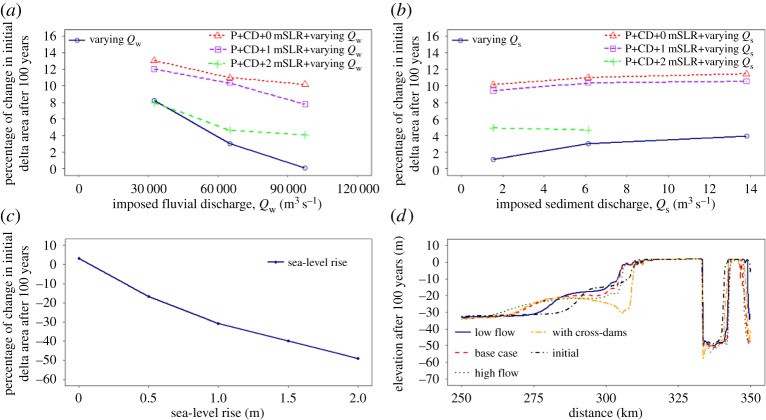
Qualitative synthesis of 100 years of modelled delta response in terms of: (*a*) sub-aerial delta area as a function of varying fluvial water discharge; (*b*) sub-aerial delta area as a function of varying fluvial sediment discharge; (*c*) sub-aerial delta area as a function of varying rates and amount of sea-level rise; and (*d*) progradation forced in response to varying fluvial water discharges and cross-dams. CD, cross-dams; P, polders; *Q*_w_, water discharge; *Q*_s_, sediment discharge; SLR, sea-level rise. (Online version in colour.)

Figure 5*d* shows that these alterations in the flow field through the delta induced differences in the pattern of simulated erosion and accretion when comparing the cross-dam and base case scenarios. As anticipated, due to the backwater effect, accretion (blue shades in figure 5*d*) increased within the distributary channels on either side of the cross-dams. The increased water discharges on the distributaries also led to channel deepening and widening (red shades in figure 5*d*) due to bed and bank erosion. Progradation of the islands on the delta front was also affected by the remote presence of the cross-dams. In particular, erosion was evident on the island fronts in this simulation, which in turn inhibited further progradation of the southern island of the delta (longitudinal section shown in figure 8*d*). The island located at the delta apex experienced less erosion. The pattern of erosion and deposition towards the sea was also influenced by the cross-dams, with more bed erosion focused in front of the southern island and more sediment deposition on either side (figure 5*d*). Due to the resistance to flow caused by the cross-dams within their distributary channel, water was diverted to flow through the other distributary channels. For those distributaries acting as main flow carrier, their mouths experienced erosion due to the higher local sediment transport capacity. An important finding, given that their design purpose is to induce accretion, is that the overall ratio of accretion and erosion over the delta was 0.97, meaning that, at the scale of the delta as a whole, the response to the introduction of cross-dams was net erosion (figure 5*d*). We discuss implications of this finding further in §4b.

### (d) Tidal delta morphology under multiple drivers of change

In this section we explore the extent to which the combined effects of multiple drivers of change—scenarios that are arguably more likely when considering real-world deltas—result in morphodynamic responses that diverge from those expected by simple summation of the responses to each individual driver acting alone. Hence, we consider the combined effects of polder-dykes, cross-dams, RSLR and varying fluvial water and sediment discharges.

#### (i) Patterns of accretion and erosion

Figure 6*a–e* shows differences in the patterns of simulated erosion and accretion between the base case scenario, in which the effects of polders and cross-dykes are excluded (figure 4*c*), versus a series of combinatorial scenarios in which the effects of polder-dykes and cross-dams interact with changing rates of sea-level rise and imposed flow and sediment discharges. Note that, in all of these scenarios, sedimentation inside the poldered area and bank erosion of the islands is excluded. Considering first the pattern of erosion and accretion over the delta in the scenario in which both polder-dykes and cross-dams are present (figure 6*a*), it is evident that the overall pattern in this case was similar to the pattern observed in the scenario with cross-dams only (figure 5*d*). However, the additional, interacting effect of polder-dykes is evident in locations far from either set of infrastructure, including at places such as in the southwest corner of the delta, where there is greater erosion on the bed of the distributaries compared to the scenario with cross-dams only. This is because of the increased sediment carrying capacity arising from the increased flow through the distributaries in the western part of the delta due to flow steering by the cross-dams, combined with the increased flow in the distributaries due to the prevention of flooding in the poldered regions.

Figure 6*b* shows the difference in the patterns of simulated erosion and accretion between the base case scenario and a scenario in which these two sets of engineering interventions interact with 2 m of RSL (with the imposed fluvial and sediment discharges set at their baseline values). As in the morphodynamic response to RSLR scenario, the impact of RSLR in terms of inducing net accretion over the bed of the distributaries clearly increased with increasing RSLR. However, what is noteworthy is that the overall accretion caused by the combination of cross-dams and polder-dykes is further enhanced by increasing RSLR (figure 7), but that zones of erosion are counteracted by the effects of the increasing RSLR (figure 6*b*). Similarly, when an increasing sediment discharge is imposed (figure 6*c*), accretion increased further and erosion decreased relative to the accretion and erosion described for the combined polder-dykes and cross-dams scenario (figure 6*a*). The increase in RSLR resulted in increased accommodation space. When combined with the increased rate of sediment supply, this resulted in increased sediment deposition to balance the accommodation space created by the RSLR. The effect of increasing sediment discharge therefore acted together with the cross-dams and polder-dykes to cause more accretion and less erosion of the distributaries than would be the case in the scenario with cross-dams and polder-dykes alone. This finding highlights how engineering structures stimulate deposition, and hence offset delta drowning, but that the efficacy of such interventions is modulated strongly by the extent to which sediment is available for deposition.

Figure 6*e* shows the morphodynamic difference in the pattern of erosion and accretion simulated between the base case scenario and a scenario in which polder-dykes and cross-dams are combined with an increased (relative to the baseline) imposed water discharge (to *Q*_w_ = 97 500 m^3^ s^−1^) and 1 m of RSLR. In this scenario, accretion decreased and erosion increased over the bed of the distributaries, while the extent of seaward deposition of sediment also increased due to the additional momentum of the flow. This response illustrates the competing interactions at play in the morphodynamic response: on the one hand the sediment transport capacity of the flow increases with increasing water discharge, while on the other hand the accommodation space and rate of sediment deposition increases with increasing RSLR. Figure 7 shows that the ratio of net accretion to erosion after 100 years of simulation time are both highly influenced by the human interventions when combined with the natural drivers. In general, the ratio of net accretion to net erosion increases due to the addition of engineering structures, but as the RSLR increases, the ratio of net accretion to net erosion increases at a greater rate than the response to RSLR alone.

#### (ii) Sub-aerial delta area

We next present variations in simulated delta area, a parameter that has obvious implications for the inhabitants of deltas and their ability to have a successful livelihood. Figure 8*a–c* shows the percentage change in the initial sub-aerial delta area after 100 years of simulation. Flooding of the deltaic floodplain occurs during the flood flow phase of the tides when the water level in the distributaries of the delta is higher due to the interaction of outgoing fluvial water and incoming tidal water discharges. The amount of sediment supply in the flood phase of the tides influences the amount of accretion and erosion over the land surface. The higher sediment supply relative to the transport capacity of the flow during flood flows leads to greater rates of accretion over the sub-aerial delta. As expected, greater rates of island bank erosion and island surface erosion occurred during the ebb flows. The rate and amount of island bank erosion increased with increasing ebb flow. In all the scenarios of varying fluvial discharge, the simulated initial sub-aerial delta area changes due to: (a) island bank erosion; (b) accretion of the land surface, which is mainly in the form of progradation of the islands into the sea; and (c) accretion at the head of the discontinuous distributaries. Most islands experienced at least some degree of erosion. Significant erosion was evident on the banks of islands at the delta apex. In contrast, islands at the delta front prograded. The footprint of the delta land area among these scenarios is not significantly different, with the difference being only in the extent of accretion and erosion and channel erosion (figures 4–6). Figure 8*a*,*b* shows that: (i) the net gain in sub-aerial delta area decreases with increasing water discharge and increases with increasing sediment discharge; and (ii) changes in land area are more sensitive to variations in fluvial water discharge than variations in sediment discharge. Similar trends are evident for the net volume change in the delta, as shown in figure 7. A water discharge with relatively high sediment supply results in a greater area and rate of accretion over the sub-aerial delta.

**Figure 9.**
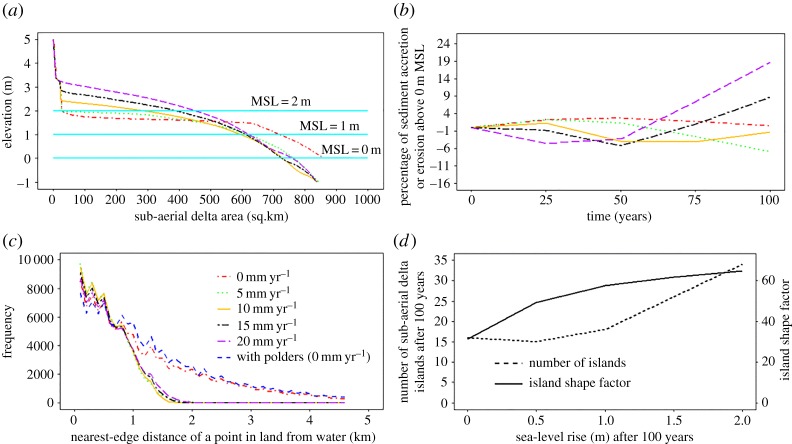
Response of the model delta to varying rates of sea-level rise: (*a*) hypsometric curve over and near the sub-aerial delta for varying sea-level rise (SLR) scenarios after 100 years; (*b*) time series of sediment accretion or erosion above the 0 m mean sea level (MSL) with respect to the initial condition for varying sea-level rise scenarios; (*c*) frequency function of nearest edge distance of a point in land from water for the modelled idealized tidal delta; and (*d*) influence of sea-level rise on number of islands and island shape factor after 100 years of simulation. (Online version in colour.)

As sea level rises, as shown in figure 8*c*, the sub-aerial delta area shrinks. The initial sub-aerial delta undergoes submergence due to increasing RSLR, leading to intense land aggradation and erosion, with the latter dominating the former during the RSLR. However, aggradation also leads to an increase in land elevation that remains above the raised mean sea level (figure 9*a*) and the amount of aggradation increases with increasing RSLR. Accretion at the delta front occurs in all the RSLR scenarios, as shown in figure 5*a*,*b*. The reduction in sub-aerial delta area implies that the rate of aggradation cannot keep pace with the rate of RSLR (figure 8*c*). With sea-level rise leading to rapid inundation of the lowland portions of the delta, but with the ‘high’ land portions growing prominently over the deltaic islands as shown by the hypsometric curves in figure 9*a*, at least 50% of the initial sub-aerial delta area remains above 2 m for the sea-level rise scenario. This is a direct result of the induced aggradation over the tidal floodplain.

We now discuss the response of the sub-aerial delta due only to the variations in the simulated engineering interventions. Individually, the polder-dykes led to a 10% increase in the sub-aerial delta as the poldered areas in the model do not undergo any accretion or erosion; however, in the real world this may not be a completely realistic assumption. This simulated increase in the area of the sub-aerial delta was completely due to the accretion focused at the delta island fronts and at the head of the intra-island channels. In the case of the cross-dam simulations, the results show that cross-dams connect islands, however after 100 morphological years about 4% of the initial sub-aerial delta area was lost. Thus although the presence of cross-dams within the distributaries of the simulated delta may promote the local development of the sub-aerial delta, this may nevertheless not result in a net gain of area across the sub-aerial delta. It is interesting to note that the effect of cross-dams on the sub-aerial delta area as reported in this paper is similar to that anticipated by Sarker *et al*. [55] based on their past experience of building cross-dams in the contemporary GBM delta.

Finally, the response of the sub-aerial delta when both natural and human drivers of change were varied is also presented. Figure 8*c* shows that the sub-aerial delta area decreased with increasing RSLR as well as increasing flow discharge and the trend remained the same even with the addition of polder-dykes and cross-dams (figure 8*a*). However, with both sea-level rise and polder-dykes, the delta area was larger than in the scenario without them. Figure 8*b* shows that the sub-aerial delta area increased with increasing fluvial sediment concentration, but not by as much as expected when both the polder-dykes and RSLR are active. However, in scenarios with polder-dykes and sea-level rise, the percentage of poldered area below MSL increased with increasing sea-level rise. In the combined scenario, accretion and erosion over the sub-aerial delta occurred only outside the poldered area. Similar to the scenario of polder-dykes, the area of accretion and erosion over the sub-aerial delta is smaller than in the base case scenario. However, the amount of erosion on the sub-aerial delta increased with increasing water discharge and RSLR and the amount of accretion increased with increasing RSLR and decreased with increasing water discharge. As the RSLR increased, the extent of the sub-aerial delta formed outside the poldered area decreased because of the increased submergence. The area and rate of accretion over the sub-aerial delta started from zero and increased with increasing sediment discharge and RSLR. Increasing RSLR therefore enhanced the effect of increasing the sediment discharge on accretion, but none of these effects were observed within the distributaries where the cross-dams were located.

#### (iii) Progradation of sub-aqueous delta

Figure 8*d* shows that the simulated progradation of the sub-aqueous delta increases with increased fluvial water discharge under relatively high sediment supply conditions. The clinoform of the delta, as well as the topset, foreset and bottom set are all clearly visible, as expected for a tidally-influenced delta [31]. Figure 8*d* shows that the simulated progradation of the delta was more sensitive to variations in water discharge than to variations in the sediment discharge, due to the tidal asymmetry near the distributary mouths. When the confined ebb flow enters the sea, because of the rapid expansion in the cross-sectional area, the jet slows down. Eventually, the sediment transport capacity of the flow decreases and starts to deposit the sediment, as described by Edmonds & Slingerland [10]. Because high water discharges have greater momentum during low tides (figure 4*d*) as compared to lower discharges, sediments are deposited at a longer distance from the mouths of the distributaries, as observed by Islam *et al*. [72]. This same effect also led to longer and flatter foresets in the scenarios with higher flow discharge and high sediment supply (figure 8*d*). However, as shown in figure 6*a–e*, under the combined influence of all drivers, the progradation of the delta was hindered by the cross-dams and the shape of the clinoform was also highly influenced by these structures (figure 8*d*).

## 4. Discussion

### (a) A transferable methodological approach?

In this study, we have demonstrated that a process-based numerical model can build an idealized mega-delta and be used to explore its response to plausible drivers of environmental change. As well as the interplay between fluvial and marine processes investigated herein, this methodology also allows for an investigation of the role of human agency. One important advantage of the adopted method is that it helps to understand the processes and mechanisms of morphodynamic change over multi-decadal timescales and under multiple drivers of change; the model is in effect used as a substitute for empirical data that is difficult to collect over the large spatial scales of real world mega-deltas. While the precise rate and magnitude of morphodynamic changes in real world deltas may not match those simulated in the idealized system investigated here, insight is likely to be afforded into the relative importance of the factors considered and their influence on delta adjustment in real-world tide-dominated deltas. This methodological approach, in which idealized ‘analogue’ morphodynamic templates are employed, is relatively new [15,27–30]. The approach has clear potential to be employed in future studies that seek to capture the intrinsic dynamics and behaviour of morphological systems (different types of delta, barrier island and lagoons, estuaries, *etc*.), as opposed to replicating specific geomorphic systems, such as the GBM or Yangtze delta.

### (b) Delta land building under environmental change

In §3, the hydrodynamic and morphodynamic response of modelled delta to individual variations in environmental change, and under multiple interacting drivers of change on tidal delta morphology, was explored. Under the combined, multiple drivers of change scenarios the response of the modelled delta is not simply the sum of the response caused by each individual factor. Instead, the results presented in §3d indicate that anthropogenic interventions affect significantly the response of tide-dominated deltas to natural drivers of environmental change such as variations in sea-level rise or the imposed flow discharge. Though the anthropogenic disturbance happens at a faster rate than the environmental change, they both act together to shape the landscape in a synergistic manner. In this sub-section we discuss how delta building processes are modulated or inhibited by these human interventions. We focus in particular on the differences in delta surface hypsometric curves (figure 9*a*) as a means of highlighting the variations in delta building induced by human agency under increasing rates of sea-level rise. These hypsometric curves provide a means to evaluate the net outcome of the trade-off between delta surface aggradation and rising sea level. Figure 9*b* shows the trajectory of natural deltaic land building process over the initial sub-aerial delta for the range of RSLR scenarios considered herein. The modelled delta begins to lose sub-aerial delta immediately under the very high imposed RSLR rates of 20 and 15 mm yr^−1^. However, after 50 years of the simulation period, as the rate of sediment supply exceeds the accommodation space created by RSLR, the state of the delta switches to being in a phase of sediment accumulation, the timing of the onset of which is a function of the RSLR rate. For example, the accumulation phase initiates at 25 years into the simulation for RSLR at 20 mm yr^−1^ compared to 50 years for the 15 mm yr^−1^ RSLR scenario. Similar trends are also evident for the RSLR rate scenarios of 10 and 5 mm yr^−1^ rate, albeit in these cases the onset of the accumulation phase is beyond the 100-year period simulated here. This implies that the higher the rate of RSLR, the faster the delta land building process, albeit the absolute rate of land building is limited by the available sediment supply.

It is also noteworthy that the nearest-edge distance decreases with increasing RSLR, except in the scenarios with polder-dykes (figure 9*c*). Thus, when polder-dykes breach, the poldered area is subject to a greater extent and increased depth of flooding because of the relatively lower land elevation. Similarly, cross-dams also affect the progradation of the islands in the delta front (figure 8*d*) and the upstream propagation of channel bed aggradation caused by RSLR (figure 6*a–e*). The results of our simulations therefore indicate that for situations in which the morphodynamic responses to sea-level rise are inhibited by the presence of engineering infrastructure, such as cross-dams and polder-dykes, the natural process of land building that would otherwise be induced by sea-level rise may be hindered.

Figure 9*d* shows that the number of delta islands grows rapidly with increasing RSLR. The submergence of land due to RSLR and the continuous processes of accretion and erosion over the sub-aerial delta result in the formation of new, smaller, islands and/or the splitting of larger land masses into a number of smaller islands. Similarly, the drainage density and perimeter of the sub-aerial delta, presented here as the island shape factor in figure 9*d*, increases with increasing RSLR. However, a key point is that these natural morphodynamic responses to RSLR are inhibited by the presence of polder-dykes because the polder-dykes affect the connectivity of the distributaries and intra-island channels and the distribution of sediment flux over the delta. When the islands are polderised, the positive effect of increasing sediment supply and RSLR on the aggradation rate is, therefore, diminished. As shown in figure 8*a*,*b*, when polder-dykes are present in a rising sea-level scenario, there is only a marginal increase in sub-aerial delta outside the poldered area, but this gain is offset against the increased risk of permanent inundation of the entire sub-aerial delta. This implies that the morphological response is strongly modulated by the presence of engineering structures, so the promotion of delta building processes requires such infrastructure to be very carefully planned.

### (c) Implications for delta management

Based on our results it is possible to infer some lessons regarding the management of tide-dominated delta systems. For example, our simulations have shown that, because of the addition of polder-dykes and cross-dams, where sufficient sediment supply is available it is possible to increase the land area of a large delta, even in the face of moderate rates of relative sea-level rise. However, it must be emphasized that in such cases the deposited sediment does not contribute to raising the land within poldered areas. Thus, a key finding of our work is the need to consider the possibility that infrastructure such as cross-dams may induce positive benefits (in terms of delta aggradation that can offset rising sea levels) locally, but that the benefits may be offset by increased erosion elsewhere, and no net gains of land occur at the delta scale.

In terms of delta management options for tide-influenced deltas at risk of delta drowning, our simulations provide insight into the relative benefits of various interventions. First, our simulations reveal that ‘do nothing’ options, for example in the case of deltas with existing polders, can lead to irreversible catastrophe, particularly if poldered areas are not elevated, because the risk of major inundation increases over time. However, the option of removing polder-dykes to revert to a more natural delta state is, in many deltas, not normally socio-economically or politically acceptable [4]. In fact, enhancing polders and building new polders is more likely under climate change and subsidence scenarios. Moreover, the option of raising or maintaining existing polder-dykes will only provide a short-term solution, and this development pathway requires an ongoing commitment to engineering maintenance and progressive upgrade. The maintenance of polder-dykes can help preserve poldered areas from erosion, but with increased fluvial water discharge and RSLR (common responses in many deltas under realistic climate change scenarios), sediment starved polder-dykes will only increase the vulnerability of protected areas to dyke failure. Such strategies also cause a ‘lock-in’ to a highly engineered situation. Hence, based on this study, we can conclude that the option of raising land and replicating the natural sedimentation response of deltas in some controlled way is (where possible) a better long-term solution.

Limited exploration of this kind of renaturalization option has begun in the context of the GBM delta, as well as some other deltas. Tidal river management with polder-dykes i.e. intentional breaching of polder-dykes to allow controlled flooding and sedimentation inside the poldered area in the GBM delta have worked in a few localized instances, but have failed elsewhere due to improper planning and implementation [32]. Also, the unplanned dyke breaching during cyclone Aila in 2009 showed the opportunity to raise the land rapidly by sedimentation [57]. There are studies that have sought to model the tidal river management required to raise the poldered areas [73], but as demonstrated herein such approaches really need to be applied at the scale of the entire delta rather than focusing on individual polders. Moreover, the high population densities in the rural GBM delta and other similar deltas, means that the social dimensions of controlled flooding require very careful consideration.

It is important to note a widespread move to much larger scale delta management such as in the Netherlands, Mekong and GBM deltas [74] and the Mississippi delta [54]. This goes hand-in-hand with increasing recognition of the multiple drivers that are impacting deltas today [75] and the need for science to provide policy relevant information at the delta scale. Hence the methods and results shown in this paper have a policy audience, and there is a need to develop this type of application.

## 5. Conclusion

This research has shown that a quantitative numerical modelling approach may be applied to develop important insights about large-scale delta morphodynamics. Our study represents the first attempt to model an idealized tidally dominated delta over the large spatial and temporal time scales that are pertinent to delta management. The method adopted here systematically analyses the influence of both natural and human drivers on delta morphodynamics. Similar approaches could in the future be adopted to explore the morphodynamic response of other delta types i.e. wave- and river-dominated, to natural environmental and human drivers of change.

All the factors considered here (fluvial water discharge, sediment discharge, relative sea-level rise, construction of polder-dykes and cross-dams), both individually and in combination, influenced tidal asymmetry (i.e. the difference in the magnitude and duration between ebb and flood tidal currents) across the modelled delta. The relative magnitude of the flood : ebb flow ratio was found to vary between 0.1 (for the high water discharge scenario) and 1.0 (for the cross-dams scenario). This is important because the tidal asymmetry and rate of sediment supply together affect residual flows, patterns of erosion and accretion, aggradation and progradation of the delta and hence the overall sub-aerial delta morphodynamics. As expected, the area of the simulated sub-aerial delta increased with increasing sediment discharge supplied from the catchment upstream, but decreased with increasing water discharge. However, delta progradation rates were more sensitive to variations in water discharge than they were to variations in sediment supply. Under all the relative sea-level rise scenarios considered here (5, 10, 15 and 20 mm yr^−1^), both the accommodation space and the rate of sediment supply varied over time and space over the delta. Overall, land tended to be lost on the landward side, whereas aggradation was focused at the delta front for all the investigated scenarios. Despite the inundation of lowland near the distributaries, aggradation enabled land building to cope with sea-level rise.

Large-scale human modifications were also found to be an important controlling factor on delta morphodynamics. For example, although the sub-aerial delta was predicted to shrink with increasing RSLR, it does not shrink when the sub-aerial delta is polderized. Indeed, the use of polder-dykes led to an increase in sub-aerial delta area over time, provided that the polder-dykes are not constrained by erosion. However, the land within the polder-dykes became more vulnerable to flooding as it lost relative elevation, increasing flood depths if and when failure occurred. Cross-dams built across a deltaic distributary to promote land accretion were also shown to accomplish their local goal, but did not always result in net land gain at the scale of the delta as a whole. This latter finding is contrary to a widespread perception of delta managers, who often assume that cross-dams produce a net gain in delta area [76], albeit there is some evidence that this perception may now be changing [55]. Also of note is the point that the combined influence of driving factors on the morphodynamic response was clearly found to not be the same as the sum of their individual effects. For example, as poldered areas lost elevation with increasing RLSR, dyke failure could result in permanent inundation of the deltaic islands, unless the poldered area was allowed to gain elevation to compensate for the rising sea level. The natural process of land building by aggradation over the sub-aerial delta during RSLR is therefore prevented by polder-dykes. Thereby while polders offer immediate benefits, over many decades they bring the increased risk of permanent inundation. While not investigated here, allowing controlled sedimentation within the polders may provide an alternative management strategy to address this adverse tendency.

The combined response of the modelled delta indicates that the interventions affect the distribution of water and sediment flux in deltas and alter the natural morphodynamic evolution of deltas. Thus, agreeing with the observations of Syvitski *et al*. [3]. Overall, this paper systematically explores the links between contemporary environmental changes and tidal delta morphodynamics over multi-decadal timescales and thus provides: (i) new guidelines to understand the factors stimulating morphodynamic change on large deltas, (ii) insight into the possible effects of future scenarios of climate, catchment management and other relevant factors on tidal delta morphology, and (iii) a basis to plan future actions to direct the geomorphological responses of large tidal deltas. Above all our simulations show that careful planning is required to ensure that human interventions that are intended to buffer against the adverse impacts of relative sea-level rise do not have potential negative consequences both locally and non-locally. This implies that evaluations of such interventions must be undertaken at the scale of the entire delta, which is a challenging task for large deltas. Our methodological approach offers a potential way to assess the implications of morphodynamic responses on deltaic systems, enriching the appraisal of large-scale delta management options

## Supplementary Material

Numerical model setup

## Data accessibility

The underlying data in this paper are available from ‘Angamuthu, Balaji, Darby, Stephen, & Nicholls, Robert. (2018). Multi-decadal morphological evolution of tidally-influenced deltas (v. 1) [Data set]. Zenodo: http://doi.org/10.5281/zenodo.1291601’. Details relating to the model set-up and the experimental scenarios can be found in the paper and its electronic supplementary material. This is an open access dataset, which permits unrestricted use, distribution, and reproduction, provided the original author and source are credited.
