# Supplementary material for: Impacts of natural and human drivers on the multi-decadal morphological evolution of tidally-influenced deltas
Source: Proc Math Phys Eng Sci. 2018 Nov 7;474(2219):20180396. doi: 10.1098/rspa.2018.0396 (PMC6283906; doi:10.1098/rspa.2018.0396)
Supplement: Numerical model setup [file rspa20180396supp1.docx]

**Supplementary material for:**

Impacts of natural and human drivers on the multi-decadal morphological evolution of tidally-influenced deltas

Angamuthu, B.,^1^ Darby, S.E.,^1^ and Nicholls, R.J.^2^

*^1^Geography and Environment, University of Southampton, SO17 1BJ UK;*

*^2^Engineering and the Environment, University of Southampton, SO17 1BJ UK.*

1. **Numerical model setup**

The two dimensional model employed herein was built in the Delft3D modelling software environment and consists of a 350km long channel, representing a river upstream of an estuary, with the channel width converging from 7km to 3km. The estuary is represented by a funnel with dimensions (180km by 120 km), which is very similar to the real world GBM delta/Meghna estuary as shown in Figure A. The model is extended in order to reduce the influence of downstream boundary conditions on the delta forming area, with the initial bathymetry employed based on the 1990s and 2000s bathymetric surveys of the lower Meghna River and Meghna estuary. The basin has a steeper bed slope near the downstream boundary to represent the steeper continental shelf. The model mesh has a resolution of 200m x 200m within the channel, estuary and in the basin for the width of the estuary. In comparison, the basin extension on either side of the estuary has a resolution of 200m x 400m near the estuary, reducing to 200m x 1600m in the outer sea. The generated model grid fulfils numerical stability requirements and therefore avoids any numerical diffusion/dispersion errors. The banks of the channel are non-erodible, whereas the banks of the estuary are erodible. The upstream river discharge and downstream water level form open boundaries in the model grid, whereas elsewhere the model grid boundaries are closed (See section 2.2 in the paper for the concept and actual values of the boundary conditions used). For the sediment transport computation we used the Van Rijn (1993) (calculates bed & suspended load) advection-diffusion equation as based on Ali et al. (2007), in which the sand and silt fractions are treated separately.

Table A shows the model parameters used for the runs in this study and summarises the basis of their selection. Hydrodynamic computations were performed using a time step of 0.25min. The initial sediment layer thickness at the bed is 40m, which is assumed to be equal to the maximum depth of the lower Meghna River.


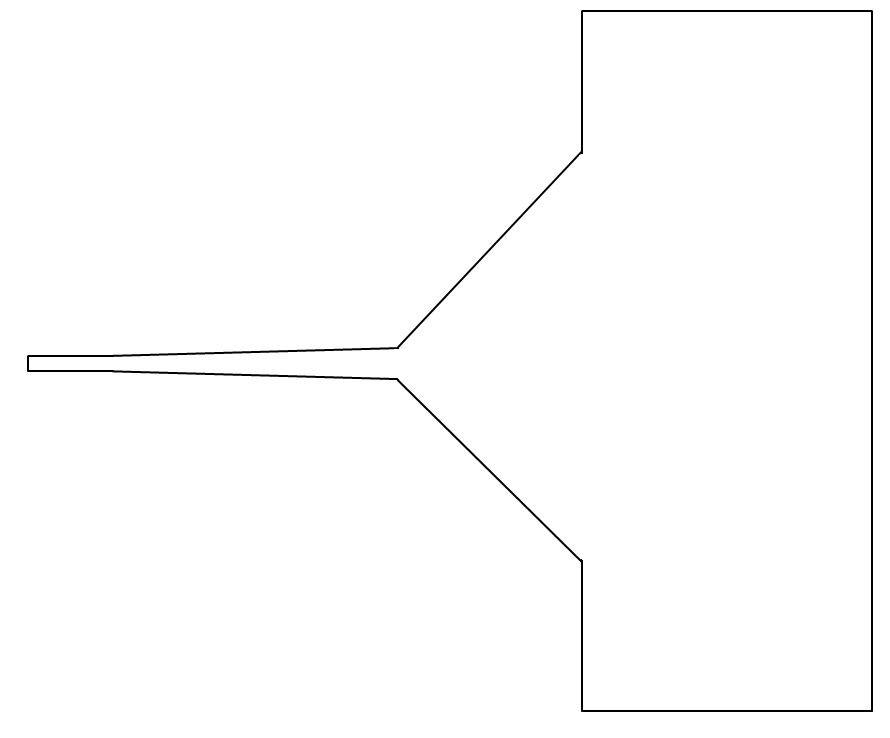


200mx 200m

200mx 1600m

200mx 800m

200mx 400m

500km

350km

Tides

River

180km

7km

3km

120km

228km

6.5m

34m

37m

150m

1000m

Mean Sea Level

FIGURE A: A sketch of the schematised 2D model (not to scale): (a) geometry (top view), and (b) initial bathymetry (longitudinal view) based on BWDB 2000s bathymetric survey of the lower Meghna river and the GBM delta/Meghna estuary.

| **Parameter** | **Basis of selection for model parameter** | **Model value** |
| --- | --- | --- |
| Initial bed composition | In mid 1980s, at 9 boring locations ([Anonymus, 1986](#_ENREF_2)), each 20m deep, in the Sandwip and Noakhali Islands of the Ganges-Brahmaputra-Meghna delta revealed sub-surface layer consisting of: 45% Sand+ 55% silt | 50% very fine sand and 50% coarse silt |
| Sediment fraction | In Padma River ([Sarker et al., 2003](#_ENREF_3)),  bed sediment D_50_  is 0.12mm (very fine sand)  [Ali et al. (2007](#_ENREF_1)) used cohesive D_50_ of 0.05mm (coarse silt) | Non-cohesive D_50_ = 0.12mm  Cohesive D_50_= 0.05mm |
| Bed slope factor for bed load transport | Longitudinal: 1  Transverse: Calibration | 1 for longitudinal and 100 for transverse |
| Spatially constant hydrodynamic roughness | Calibration^#^. Assuming bed with dunes and no vegetation | Manning’s n = 0.055 |
| Dry cell erosion factor | Calibration | 1 |
| Sediment transport formula | Van Rijn 1993 (calculates bed & suspended load) advection-diffusion equation used | Van Rijn 1993 |
| Horizontal eddy viscosity^#^ | Calibration | 1m^2^/s |
| **For cohesive sediments** | | |
| Critical shear stress for erosion | Calibration and [Ali et al. (2007](#_ENREF_1)) used 0.2 N/m^2^ for D_50_ = 0.05mm | 0.1 N/m^2^ |
| Critical shear stress for sedimentation | Calibration and [Ali et al. (2007](#_ENREF_1)) used 0.1 N/m^2^ for D_50_ = 0.05mm | 0.1N/m^2^ |
| Fall velocity | 2.25mm/s for 0.05mm based on Stoke’s law | 2.25mm/s |
| Horizontal eddy diffusivity | Calibration | 10m^2^/s |

Table A: Model parameters for runs in this study

**References**

ALI, A., MYNETT, A. E. & HAMMADUL AZAM, M. 2007. Sediment dynamics in the Meghna estuary, Bangladesh: A model study. *Journal of Waterway Port Coastal and Ocean Engineering-Asce,* 133**,** 255-263.

ANONYMUS, N. N. 1986. Sandwip Crossdam. Delft: TUDelft.

SARKER, M. H., HUQUE, I., ALAM, M. & KOUDSTAAL, R. 2003. Rivers, chars and char dwellers of Bangladesh. *International Journal of River Basin Management,* 1**,** 61-80.

VAN RIJN, L. C. 1993. *Principles of Sediment Trasnport in Rivers, Estuaries and Coastal Seas,* Amsterdam, Aqua Publisher.
